# Weekday and Weekend Differences in Eating Habits, Physical Activity and Screen Time Behavior among a Sample of Primary School Children: The “Seven Days for My Health” Project

**DOI:** 10.3390/ijerph19074215

**Published:** 2022-04-01

**Authors:** Francesco Esposito, Francesco Sanmarchi, Sofia Marini, Alice Masini, Susan Scrimaglia, Emanuele Adorno, Giorgia Soldà, Fabrizio Arrichiello, Filippo Ferretti, Marilisa Rangone, Francesca Celenza, Emilia Guberti, Domenico Tiso, Stefania Toselli, Antonello Lorenzini, Laura Dallolio, Rossella Sacchetti

**Affiliations:** 1 Department of Biomedical and Neuromotor Science, University of Bologna, 40126 Bologna, Italy; francesco.esposito32@studio.unibo.it (F.E.); francesco.sanmarchi@studio.unibo.it (F.S.); susan.scrimaglia@studio.unibo.it (S.S.); emanuele.adorno@studio.unibo.it (E.A.); giorgia.solda@studio.unibo.it (G.S.); fabrizio.arrichiello@studio.unibo.it (F.A.); marilisa.rangone@studio.unibo.it (M.R.); stefania.toselli@unibo.it (S.T.); antonello.lorenzini@unibo.it (A.L.); laura.dallolio@unibo.it (L.D.); 2 Department for Life Quality Studies, University of Bologna, 47921 Rimini, Italy; sofia.marini2@unibo.it; 3 Department of Public Health, Bologna Health Trust, 40121 Bologna, Italy; filippo.ferretti@ausl.bologna.it (F.F.); francesca.celenza@ausl.bologna.it (F.C.); 4 Hygiene and Nutrition Service, Department of Public Health, 40121 Bologna, Italy; emilia.guberti@gmail.com; 5 Hospital Villa Maria, 47921 Rimini, Italy; dottortiso@gmail.com; 6 Department of Education Studies “Giovanni Maria Bertin”, University of Bologna, 40126 Bologna, Italy; rossella.sacchetti@unibo.it

**Keywords:** KIDMED index, diet quality, sedentary behavior, children, school canteen, public health, physical activity

## Abstract

Background: Healthy eating and active lifestyle habits are essential for a child’s development, wellbeing, and health. School setting and family environment play a crucial role in shaping these habits and this could be reflected in different behavior patterns during weekdays and weekends. Methods: We investigated primary school children’s lifestyle habits through a cross-sectional analysis of 428 Italian primary school children, with a mean age of 8.99 years (±1.43). Data were collected from May to June 2017 using a weekly diary to assess children’s lifestyles. Results: Children who eat their morning snack and lunch at school three or more times during the weekdays were 5.47 times more likely (95% CI 3.02, 10.2) to consume adequate snacks and 7.79 times more likely (95% CI 4.43, 14.5) to have adequate meals than those who did not. Conclusion: Consumption of vegetables, lunch, and snacks are significantly more adequate during the weekdays as compared to the weekends. Physical activity levels did not differ between weekdays and weekends. Moreover, children spent more time engaged in physical activities than in front of a screen during both the weekdays and the weekends. The present results are good indicators of the importance of the school canteen in defining correct eating habits. Family-based and school-based interventions could represent valuable integrative strategies for promoting a healthy lifestyle in children.

## 1. Introduction

Improving diet quality and overall lifestyle is a key health promotion strategy [1]. For decades, the Italian healthcare system has implemented numerous public health campaigns to promote a healthy lifestyle (i.e., day-to-day behaviors and functions including physical activity and diet) [2]. Even if the public health campaigns have been an effective tool to improve the overall wellbeing of individuals, [3,4] most of the Italian population still does not follow an optimal lifestyle, has an unbalanced diet, and does little physical activity (PA) [5]. Even children are affected by this trend, with a significant percentage of the population presenting overweight/obesity [6], albeit with marked subnational differences [7] and low levels of PA [8], one of the lowest at international level [9,10].

Although children’s activity follows many repeated patterns and habitual behaviors, the differences between the weekend habits compared to those of the week could be associated with different factors. It has been shown, both through questionnaires and through objective measurements (accelerometer), that children have lower levels of PA on the weekend than on midweek days [11,12]. Moreover, the weekends are characterized by less adherence to an adequate diet; for example, the literature reports that eating out is more common on weekends than on weekdays, and this leads to increased discretionary calorie intake from energy-dense, nutrient-poor foods [13,14]. Furthermore, during the weekdays, a variable number of meals are provided by the school canteen. In this context, school plays a critical role. Specifically, through canteens, schools provide adequate meals to children regardless of their socioeconomic status [15]. Moreover, it provides nutritional education that can also be followed in the home environment [16].

Plenty of studies have investigated the differences between the weekend and weekday dietary patterns [17,18,19,20,21,22,23,24] and PA levels [25,26] in children, showing less healthy dietary intakes and lower levels of PA during weekends in comparison to weekdays. To the best of our knowledge, no studies were conducted focusing on the Italian primary school population and analyzing not only dietary patterns but also PA levels, screen time, and the influence of the school canteen.

Built on previous literature, this study first examined weekend-weekday differences in diet, PA levels, and sedentary behavior. The second aim of the present study was to analyze the impact of the school canteen on meal adequacy during the week among Italian primary school children using data from a local representative survey.

## 2. Materials and Methods

### 2.1. Study Design and Participants

This cross-sectional study was conducted among a sample of children enrolled in the “Seven days for my health” project, in the primary schools of Calderara di Reno, in the province of Bologna in Emilia Romagna Region, Northern Italy. The Bioethics Committee of the University of Bologna approved the “Seven days for my health project” on 30 June 2016.

The study was conducted following the Declaration of Helsinki.

Children from the 1st to the 5th grades were enrolled in the project. Schools did not have an internal canteen but received meals from external food establishments which provided lunches but also the morning snacks. No further inclusion/exclusion criteria have been adopted. The research team collected written parents’ informed consent to participate in the study. This study was designed following the Strengthening the Reporting of Observational Studies in Epidemiology (STROBE) reporting guidelines [27].

### 2.2. Study Variables

Researchers collected data from May to June 2017 administering the ‘Seven days for my health’ diary authored by Domenico Tiso. This diary assesses children’s lifestyle and dietary habits on a weekly basis [28]. The data collection process was completely anonymous, and the researchers evaluated each diary for completeness and accuracy before the analysis. Notably, children compiled the diary under the supervision of a parent or a caregiver, which helped reduce the risk of bias. 

Body Mass Index (BMI) was used to assess the weight status of each participant according to Cole cut off values by sex and age [29,30].

The “Seven days for my health” diary was constructed in five different sections [31].

Section 1: nationality and anthropometric characteristics of the child: age, gender, height, and weight. Height and weight were measured by the researchers.

Section 2: children’s weekly physical activity and daily screen time. 

Section 3 and Section 4: parents or legal guardians’ weight, height, education level, occupation, physical activity, and dietary habits. 

Section 5: children’s daily dietary habits (specifically, breakfast, mid-morning snack, lunch, mid-afternoon snack and dinner).

We dichotomized counting variables (the number of times in a week children eat lunch at school and number of times in a week children eat snacks at school) using 50% of the maximum possible value as the cut off (1 if >50% and 0 if ≤50%). 

### 2.3. Lifestyle Assessment

We evaluated children’s lifestyles by investigating different factors. We calculated the adherence to the Mediterranean diet using the Diet Quality Index for Children and Adolescents (KIDMED) score (0 to 12) [32,33]. We classified the individuals in three distinct categories based on their KIDMED index [34]: (1) 8–12 high; (2) 4–7 medium, and (3) 0–3 poor. We counted the number of meals consumed at school (1) or elsewhere (0) for each meal. We labeled the average week/weekdays/weekend meal as adequate (1) or inadequate (0) based on guidelines for healthy meals and diet [35]. 

The intake of fruit and vegetables was calculated on the basis of the 5 recommended portions (80 g each or 400 g in total) [36].

### 2.4. Breakfast Composition Qualitative Assessment

The qualitative assessment of breakfast composition was conducted using a specially designed score. The scoring criteria were designed by an Italian research group to use together with the diary [37]. The usage of this score to assess if breakfast is a well-balanced meal is supported by the European recommendations of a healthy diet and meal composition presented in the form of a healthy eating plate. Breakfast composition was rated according to the content of water (+1), carbohydrates (+1), proteins or dairy products (+1), fiber (+1), vitamins/minerals (+1), and free sugars (−1), assuming a well-balanced breakfast if scoring ≥ 3 points (each point in a different category) and containing a source of proteins or dairy products and carbohydrates. 

### 2.5. Morning Snack Qualitative Assessment 

We considered a snack to be adequate if it contains fruits or yogurt and non-adequate with other type of snack or skipped the snack [38].

### 2.6. Lunch/Dinner Composition Qualitative Assessment 

We considered a lunch and dinner to be adequate if it contained carbohydrates (pasta, rice, soups, bread, potatoes), proteins (meat, fish, eggs, cheese, legumes), and vegetables (raw vegetables, cooked vegetables, vegetable soup) [39].

### 2.7. Physical Activity Levels and Sedentary Behavior

Children’s PA levels were calculated asking how many minutes per day in a week were spent doing sport or recreational-motor activity. In consideration of children’s screen time, we assessed the time spent (minutes) on television (TV) and personal computer (PC) or videogames, recording the time of use during the day. 

### 2.8. Statistical Analysis

Continuous variables are reported using mean and standard deviation (±SD) and categorical variables using absolute and relative frequencies. The normal distribution of the selected variables was assessed with the Shapiro–Wilk test and their distribution was investigated using density graphs.

Univariate analysis was performed to assess the differences between weekend and weekday habits and between meal adequacy in children who eat school-provided meals and those who do not eat school-provided meals. Student t-test was used to compare means of independent groups, Wilcoxon signed-rank test to compare ordinal variables in dependent groups, McNemar’s test to compare dichotomous variables in dependent groups, and Chi-Square test to compare dichotomous variables in independent groups.

The associations between dichotomous variables and predictors were assessed with a multiple logistic regression model with backward stepwise selection. Results from logistic regression were reported as odds ratio (OR) and 95% CI. The significance level was set as *p* < 0.05. No questionnaires were excluded from the analysis because there were no missing data among the analyzed variables.

All analyses were carried out using R version 4.1.2 (R Project for Statistical Computing) [40].

## 3. Results

The study population consisted of 428 children, of which there were 235 (54.9%) girls and 193 (45.1%) boys, aged 6 to 10 years (mean 8.99 ± 1.43). The majority of the population had a normal weight (*n* = 346; 80.8%) and the remaining part had overweight/obesity (*n* = 82; 19.2%), according to Cole cut off values by sex and age. Out of the 428 children included in the study, 427 had breakfast every day of the week, 371 ate a snack in the morning during the weekdays, and 265 during the weekends, and every participant stated that they had lunch and dinner every day of the week. More than half of the individuals have lunch at the school canteen three times or more during the weekdays (*n* = 337; 78.7%). Detailed population characteristics and weekly habits are summarized in Table 1. 

### 3.1. Weekdays vs. Weekends

Table 2 shows the comparison between weekday lifestyle habits and weekend ones. 

KIDMED index was significantly higher (*p* < 0.001) on the weekdays (4.48 ± 1.80) compared to the weekend days (3.95 ± 1.89). Breakfast adequacy showed no significant changes between weekdays and weekends (*p* = 0.628), while snacks (*p* < 0.001) and lunches (*p* < 0.05) were more adequate during the weekdays than the weekend. Dinners were more appropriate during the weekend than during the weekdays (*p* < 0.001) and combined fruit and vegetable consumption was higher during the weekdays than the weekend (*p* < 0.001). Specifically, fruit consumption was higher during the weekend (*p* < 0.001), while vegetable consumption was higher during the weekdays (*p* < 0.001). 

Considering PA and sedentary behaviors, the average daily physical activity level did not statistically differ between weekdays and weekends (*p* = 0.790), while screen time is higher during the weekdays (83.13 ± 72.93 min) compared to the weekend (*p* < 0.001). Specifically, TV time decreased during the weekend (*p* < 0.001), while PC/videogame time remained unchanged (*p* = 0.728).

### 3.2. School-Provided Meals

**Figure 1:**
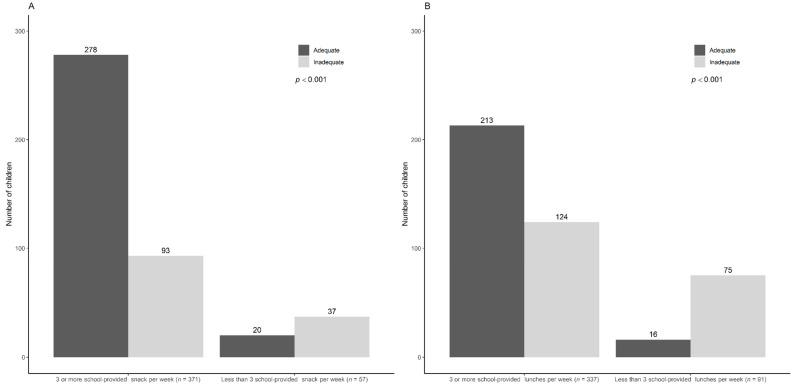
School canteen’s influence Monday to Friday. (**A**) Snacks. (**B**) Lunch.

Univariate analysis (Figure 1, Tables S1 and S2) and logistic regression models (Table 3) showed the impact of school canteens on meal adequacy. 

The results showed that children who ate their morning snack at school three or more times during the week were 5.47 times more likely (95% CI 3.02, 10.2) to consume adequate snacks than those who did not. Logistic regression reports similar results for lunch, with a 7.79 (95% CI 4.43, 14.5) times greater probability to have adequate meals for children that consumed lunch three or more times a week at school. Notably, older children were associated with a reduction in the likelihood of having adequate snacks/lunches, while gender and weight status did not affect those meal choices. These results are summarized in Table 3.

## 4. Discussion

This cross-sectional study investigated primary school children’s lifestyle habits (diet, PA and sedentary behavior) during the weekdays and the weekends and analyzed the impact of school canteens on weekday meal adequacy. These topics have been previously investigated by other authors [17,18,19,20,21,22,23,24,25,41]. However, although plenty of literature investigated Italian primary school children’s lifestyle habits, to the best of our knowledge, no studies were focused on weekday and weekend differences [7,42,43].

Among the demographic factors, it is important to describe the children’s weight distribution. In our sample, 19.2% of the children presented an overweight/obese condition, a percentage that differs from the one reported by the Italian epidemiological surveillance systems (OKkio alla SALUTE) which showed that 26.4% of children of the Emilia-Romagna region had an overweight or obese condition in 2019 [10]. This could be linked to the higher than average PA and the lower than average screen time levels reported in our sample compared to the ones shown in the “Okkio alla Salute” report [10]. On the contrary, our sample’s KIDMED index was aligned with other Italian primary school children’s dietary assessments, with only 35.5% of individuals reporting poor adherence to the Mediterranean Diet [44]. Focusing on the specific meals, our results showed that 51.2% of our sample consumed adequate breakfasts on a weekly basis. Similarly, just over half of the children (50.2%) had adequate snacks during the week. On the other hand, over the entire week, only a small portion of the other two main meals met the requirements to be classified as adequate, with only 36.9% of children reporting adequate lunches and 10.5% reporting adequate dinners. These results could be explained by an important factor. The 78.7% of our sample consumed at least three lunches a week at the school canteen which offered vegetables and fruits as essential components of the meal making it easier to achieve the adequacy of lunch than dinner.

We analyzed the lifestyle differences between weekdays and weekends using univariate analysis and logistic regressions. The significantly higher KIDMED index during the weekdays compared to weekends could be linked to the impact of the school canteen and school-provided meals (snacks and lunches), which are designed to provide the right amount of micro and macronutrients. Specifically, breakfast is the most consistent meal across the week. This finding confirmed the one reported by OKkio alla SALUTE where it is shown that 44.3% of children did not meet the requirements for an adequate breakfast [10]. In contrast with other meals, dinner was significantly more adequate during the weekends compared to the workdays. This could be explained by the reduced amount of time parents have for food preparation during workdays, as confirmed by other studies [45]. Notably, the stark difference (*p* < 0.001) between weekend and weekday fruit and vegetable consumption is determined by a lower vegetable consumption during the weekend. This finding could be linked to the influence of the school canteen that ensures an adequate intake of vegetables and other types of food [46]. Moreover, our results showed higher consumption of fruit during the weekend, while vegetables are eaten more during the week. Again, this could be a byproduct of the school canteen’s influence [47] and could also be linked to the fact that children tend to prefer fruits over vegetables [48].

Other than dietary habits, screen time differed when comparing weekends and weekdays. 

In consideration of PA and sedentary behavior, the total PA levels did not statistically differ between weekdays and weekends, while screen time was higher during weekdays. Specifically, TV time decreased during the weekend (*p* < 0.001), while PC/videogame time remained unchanged (*p* = 0.728). On a daily average, children engaged in PA for a comparable amount of time during weekdays (90.29 ± 53.53 min) and weekends (91.36 ± 89.43 min). Notably, children spent more time engaged in PA than in front of a screen during both the weekdays and the weekends. This finding could partially explain the fact that our sample was characterized by better weight status in comparison to the population of the Emilia Romagna region [10].

Although on a weekly basis our sample’s screen time was lower than the one in comparable samples [10], it was significantly higher during the weekdays (83.13 ± 72.93 min) as compared to the weekend (69.93 ± 78.13 min). This difference was determined by the higher time spent watching TV during the weekdays. This finding could be explained by the fact that on weekends parents spend more time with their children doing recreational activities, as other studies have shown [49]. PA’s and sedentary time differences between weekdays and weekends are aligned with the results reported in the recent literature [11,12,50].

One of the main reasons why certain meals were more adequate during the weekdays could be the presence of school-provided meals. The logistic regressions’ results showed that children that consumed three or more school-provided lunches per week had a higher probability (7.79; 95% CI 4.43, 14.5) to consume adequate lunches on average during the weekdays. The same was true for snacks. These are good indicators of the importance of the school canteen in determining correct eating habits. Plenty of literature highlighted the positive impact of the school canteen on children’s diet [51]. Specifically, in Italy, school-provided meals have to meet certain safety standards, provide adequate nutrition, and ensure the usage of quality ingredients and taste [47].

Notably, gender and weight status did not play a role in determining meal adequacy during the weekdays. On the other hand, older age was associated with a lower chance of consuming adequate snacks (0.77; 95% CI 0.66, 0.90) and lunches (0.84; 95% CI 0.73, 0.98) during the weekdays. This trend has been studied by many authors [52,53]. Younger children were more likely to try different types of food and older ones were more likely to be fussy [31,53]. Similarly, younger children were more likely to finish their meals than older children [53].

With the present obesity pandemic and the number of young people in Italy being overweight and inactive continuing to grow, any measures that may help in facilitating healthy food choices deserve to be put into consideration. School-based and family-based interventions that are aimed at creating a healthier environment that enables and facilitates children and adolescents to make healthier decisions are a fundamental path to be further explored. Most school-based interventions aimed at promoting healthy lifestyles in children focused only on the school setting. However, recent literature found that multicomponent interventions involving not only school but also family are likely to be most effective [54]. In light of this, family-based interventions could represent valuable integrative strategies for promoting a healthy lifestyle in children. 

This study has several limitations: (i) in this study, we grouped “Saturday” and “Sunday” together, which could determine the overlook of the distinctive patterns on each of these two weekend days (An and colleagues reported that weekend energy intake was higher on Saturday compared to Sunday) [55]. (ii) We did not take into account the seasonality of lifestyle habits. Specifically, the questionnaires were filled in from May to June, and it is possible that during the colder months of the year children had different diet and lifestyle habits. (iii) PA assessment was based on a survey and not on objective measures (e.g., accelerometer) and this could have led to PA misreporting. However, Burchartz and colleagues recently stated that PA levels assessed using accelerometers and surveys are comparable [56]. (iv) We used screen time as a proxy of sedentary behavior, and this could have led to an underestimation of inactive states.

Although the population group is limited to a specific Italian region, the sample is sufficient enough to determine relevant conclusions. Thus, the reported results could be extrapolated to a larger European context.

## 5. Conclusions

The “Seven days for My Health” project highlighted the existing weekday and weekend differences in children’s eating and physical activity and screen time behaviors among a sample of primary school children. 

Consumption of vegetables, adequate lunch, and snacks are significantly more adequate during the weekdays as compared to the weekends. 

Generally, the KIDMED index was higher during the weekdays compared to weekends. These results could be linked to the impact of the school canteen and school-provided meals to ensure an adequate intake of vegetables and other types of food. Moreover, children that consumed three or more school-provided lunches per week had a higher probability of consuming adequate lunches on average during the weekdays.

The present results are good indicators of the importance of the school canteen in determining correct eating habits. 

Although children in our sample spent more time engaged in PA than in front of a screen during both the weekdays and the weekend, the higher levels of PA during weekends than on the weekdays suggest the need to implement school-based PA programs to enhance the PA levels during weekdays. During the school day, there are many opportunities to increase levels of PA with numerous opportunities to engage students in different places and times. For this reason, school has a predominant role in influencing children’s eating and physical activity behaviors.

In conclusion, family-based and school-based interventions could represent valuable integrative strategies for promoting a healthy lifestyle in children. 

## Figures and Tables

**Table 1:** Population characteristics and weekly habits.

Population Characteristics	*n* = 428
Males	193 (45.1%)
Females	235 (54.9%)
Age (years)	8.99 (1.43)
Normal weight (*n*; %)	346 (80.8%)
Overweight/Obesity (*n*; %)	82 (19.2%)
Eating habits	
KIDMED Index, mean (SD)	4.20 (1.83)
KIDMED Index category	
Low (*n*; %)	152 (35.5%)
Medium (*n*; %)	257 (60.0%)
High (*n*; %)	19 (4.4%)
Adequate Breakfast	
No	209 (48.8%)
Yes	219 (51.2%)
Adequate Snacks	
No	213 (49.8%)
Yes	215 (50.2%)
Adequate Lunch	
No	270 (63.1%)
Yes	158 (36.9%)
Adequate Dinner	
No	383 (89.5%)
Yes	45 (10.5%)
Fruit and vegetable consumption per day (portions), mean (SD)	2.09 (0.89)
Fruit consumption per day (portions), mean (SD)	1.13 (0.64)
Vegetable consumption per day (portions), mean (SD)	0.96 (0.46)
Physical activity and sedentary behavior	
Daily screen time, mean (minutes, SD)	79.36 (67.26)
Daily PC/videogames time, mean (minutes, SD)	23.21 (35.28)
Daily TV time (minutes, SD)	55.45 (49.22)
Daily PA time, mean (minutes, SD)	90.60 (54.10)

Standard Deviation (SD).

**Table 2:** Univariate analysis.

Characteristic	Weekdays, *n* = 428	Weekend Days, *n* = 428	*p*-Value
Eating habits			
KIDMED Index (mean, SD)	4.48 ± 1.80	3.95 ± 1.89	<0.001
KIDMED Category			<0.001
Low (*n*; %)	125 (29%)	186 (43%)	
Medium (*n*; %)	282 (66%)	221 (52%)	
High (*n*; %)	21 (4.9%)	21 (4.9%)	
Adequacy Breakfast			0.628
No	209 (49%)	208 (49%)	
Yes	219 (51%)	220 (51%)	
Adequate Snacks			<0.001
No	130 (30%)	333 (78%)	
Yes	298 (70%)	95 (22%)	
Adequate Lunch			0.008
No	199 (46%)	290 (68%)	
Yes	229 (54%)	138 (32%)	
Adequate Dinner			<0.001
No	361 (84%)	326 (76%)	
Yes	67 (16%)	102 (24%)	
Fruit and vegetables (portions), mean (SD)	2.33 ± 0.91	1.49 ± 1.19	<0.001
Fruit (portions), mean (SD)	1.24 ± 0.65	1.69 ± 1.70	<0.001
Vegetables (portions), mean (SD)	1.08 ± 0.50	0.64 ± 0.62	<0.001
Physical activity and sedentary behavior			
Screen time (minutes, SD)	83.13 ± 72.93	69.93 ± 78.13	<0.001
PC/videogames (minutes, SD)	24.10 ± 37.70	23.42 ± 44.42	0.728
TV (minutes, SD)	59.03 ± 54.42	46.50 ± 57.84	<0.001
Time spent in PA (minutes, SD)	90.29 ± 53.53	91.36 ± 89.43	0.790

**Table 3:** Logistic regression models.

	Snack Adequate			Lunch Adequate		
	OR	95% CI	*p*-Value	OR	95% CI	*p*-Value
Age (years)	0.77	0.66, 0.90	0.001	0.84	0.73, 0.98	0.023
Males	—	—		—	—	
Females	1.15	0.74, 1.78	0.5	1.39	0.92, 2.12	0.12
Normal weight	—	—		—	—	
Obese/overweight	1.31	0.75, 2.36	0.4	1.12	0.66, 1.90	0.7
Less than 3 school-provided snacks per week	—	—				
3 or more school-provided snacks per week	5.47	3.02, 10.2	<0.001			
Less than 3 school-provided lunches per week				—	—	
3 or more school-provided lunches per week				7.79	4.43, 14.5	<0.001

## Data Availability

The data presented in this study are available on request from the corresponding author. The data are not publicly available due to ethical and privacy reasons.

## Supplementary Materials

The following supporting information can be downloaded at: https://www.mdpi.com/article/10.3390/ijerph19074215/s1.

## References

[1] Bull F.C., Al-Ansari S.S., Biddle S., Borodulin K., Buman M.P., Cardon G., Carty C., Chaput J.-P., Chastin S., Chou R. (2020). World Health Organization 2020 guidelines on physical activity and sedentary behaviour. Br. J. Sports Med..

[2] Ministry of Health (2015). Proper Nutrition Campaign—Eat Healthy, Invest in Health. https://www.salute.gov.it/portale/expo2015/dettaglioCampagneExpo2015.jsp?id=96.

[3] Bradley J., Gardner G., Rowland M.K., Fay M., Mann K., Holmes R., Foster E., Exley C., Bosco A.D., Hugueniot O. (2020). Impact of a health marketing campaign on sugars intake by children aged 5–11 years and parental views on reducing children’s consumption. BMC Public Health.

[4] Khow Y.Z., Lim T.L.Y., Ng J.S.P., Wu J., Tan C.S., Chia K.S., Luo N., Seow W.J. (2021). Behavioral impact of national health campaigns on healthy lifestyle practices among young adults in Singapore: A cross-sectional study. BMC Public Health.

[5] EpiCentro Surveillance Passi. https://www.epicentro.iss.it/passi/.

[6] EpiCentro OKkio Alla Salute National Survey 2019: National Data. https://www.epicentro.iss.it/okkioallasalute/indagine-2019-dati.

[7] EpiCentro OKkio Alla SALUTE: I Risultati Dell’indagine 2019 in Emilia-Romagna. https://www.epicentro.iss.it/okkioallasalute/indagine-2019-report-emilia-romagna.

[8] Konstabel K., Veidebaum T., Verbestel V., Moreno L.A., Bammann K., Tornaritis M., Eiben G., Molnár D., Siani A., Sprengeler O. (2014). Objectively measured physical activity in European children: The IDEFICS study. Int. J. Obes..

[9] Aubert S., Brazo-Sayavera J., González S.A., Janssen I., Manyanga T., Oyeyemi A.L., Picard P., Sherar L.B., Turner E., Tremblay M.S. (2021). Global prevalence of physical activity for children and adolescents; inconsistencies, research gaps, and recommendations: A narrative review. Int. J. Behav. Nutr. Phys. Act..

[10] Steene-Johannessen J., Hansen B.H., Dalene K.E., Kolle E., Northstone K., Møller N.C., Grøntved A., Wedderkopp N., Kriemler S., Page A.S. (2020). Variations in accelerometry measured physical activity and sedentary time across Europe—Harmonized analyses of 47,497 children and adolescents. Int. J. Behav. Nutr. Phys. Act..

[11] An M., Chen T., Zhou Q., Ma J. (2021). Paternal and maternal support of moderate-to-vigorous physical activity in children on weekdays and weekends: A cross-sectional study. BMC Public Health.

[12] Brazendale K., Beets M.W., Armstrong B., Weaver R.G., Hunt E.T., Pate R.R., Brusseau T.A., Bohnert A.M., Olds T., Tassitano R.M. (2021). Children’s moderate-to-vigorous physical activity on weekdays versus weekend days: A multi-country analysis. Int. J. Behav. Nutr. Phys. Act..

[13] Orfanos P., Naska A., Trichopoulos D., Slimani N., Ferrari P., van Bakel M., Deharveng G., Overvad K., Tjønneland A., Halkjær J. (2007). Eating out of home and its correlates in 10 European countries. The European Prospective Investigation into Cancer and Nutrition (EPIC) study. Public Health Nutr..

[14] Yang P.H.W., Black J.L., Barr S.I., Vatanparast H. (2014). Examining differences in nutrient intake and dietary quality on weekdays versus weekend days in Canada. Appl. Physiol. Nutr. Metab..

[15] Moore G.F., Murphy S., Chaplin K., Lyons R.A., Atkinson M., Moore L. (2014). Impacts of the Primary School Free Breakfast Initiative on socio-economic inequalities in breakfast consumption among 9–11-year-old schoolchildren in Wales. Public Health Nutr..

[16] Finch M., Sutherland R., Harrison M., Collins C. (2006). Canteen purchasing practices of year 1-6 primary school children and association with SES and weight status. Aust. N. Z. J. Public Health.

[17] Rothausen B.W., Matthiessen J., Hoppe C., Brockhoff P.B., Andersen L.F., Tetens I. (2012). Differences in Danish children’s diet quality on weekdays v. weekend days. Public Health Nutr..

[18] Rothausen B.W., Matthiessen J., Andersen L.F., Brockhoff P.B., Tetens I. (2013). Dietary patterns on weekdays and weekend days in 4–14-year-old Danish children. Br. J. Nutr..

[19] Haines P.S., Hama M.Y., Guilkey D.K., Popkin B.M. (2003). Weekend eating in the United States is linked with greater energy, fat, and alcohol intake. Obes. Res..

[20] Hanson K.L., Olson C.M. (2013). School meals participation and weekday dietary quality were associated after controlling for weekend eating among U.S. school children aged 6 to 17 years. J. Nutr..

[21] Svensson A., Larsson C., Eiben G., Lanfer A., Pala V., Hebestreit A., Huybrechts I., Fernández-Alvira J.M., Russo P., Koni A.C. (2014). European children’s sugar intake on weekdays versus weekends: The IDEFICS study. Eur. J. Clin. Nutr..

[22] Yang H.W. (2013). A Temporal Analysis of Canadian Dietary Choices Using the Canadian Community Health Survey Cycle 2.2: Does Nutrient Intake and Diet Quality Vary on Weekends Versus Weekdays?. Doctoral Dissertation.

[23] Dutch D.C., Golley R.K., Johnson B.J. (2021). Diet Quality of Australian Children and Adolescents on Weekdays versus Weekend Days: A Secondary Analysis of the National Nutrition and Physical Activity Survey 2011–2012. Nutrients.

[24] Hart C.N., Raynor H.A., Osterholt K.M., Jelalian E., Wing R.R. (2011). Eating and activity habits of overweight children on weekdays and weekends. Int. J. Pediatric Obes..

[25] Foweather L., Knowles Z., Ridgers N.D., O’Dwyer M.V., Foulkes J.D., Stratton G. (2015). Fundamental movement skills in relation to weekday and weekend physical activity in preschool children. J. Sci. Med. Sport.

[26] Wang C., Chen P., Zhuang J. (2013). A National Survey of Physical Activity and Sedentary Behavior of Chinese City Children and Youth Using Accelerometers. Res. Q. Exerc. Sport.

[27] Von Elm E., Altman D.G., Egger M., Pocock S.J., Gøtzsche P.C., Vandenbroucke J.P. (2007). Strengthening the reporting of observational studies in epidemiology (STROBE) statement: Guidelines for reporting observational studies. BMJ.

[28] Tiso D., Baldini M., Piaggesi N., Ferrari P., Biagi P., Malaguti M., Lorenzini A. (2010). “7 days for my health”: A new tool to evaluate kids’ lifestyle. Agro Food Ind. Hi-Tech.

[29] Cole T.J., Lobstein T. (2012). Extended international (IOTF) body mass index cut-offs for thinness, overweight and obesity. Pediatric Obes..

[30] Cole T.J., Flegal K.M., Nicholls D., Jackson A.A. (2007). Body mass index cut offs to define thinness in children and adolescents: International survey. BMJ.

[31] Sanmarchi F., Esposito F., Marini S., Masini A., Scrimaglia S., Capodici A., Arrichiello F., Ferretti F., Rangone M., Celenza F. (2022). Children’s and Families’ Determinants of Health-Related Behaviors in an Italian Primary School Sample: The “Seven Days for My Health” Project. Int. J. Environ. Res. Public Health.

[32] Idelson P.I., Scalfi L., Valerio G. (2017). Adherence to the Mediterranean Diet in children and adolescents: A systematic review. Nutr. Metab. Cardiovasc. Dis..

[33] Štefan L., Prosoli R., Juranko D., Čule M., Milinović I., Novak D., Sporiš G. (2017). The Reliability of the Mediterranean Diet Quality Index (KIDMED) Questionnaire. Nutrients.

[34] Serra-Majem L., Ribas L., Ngo J., Ortega R.M., García A., Pérez-Rodrigo C., Aranceta J. (2004). Food, youth and the Mediterranean diet in Spain. Development of KIDMED, Mediterranean Diet Quality Index in children and adolescents. Public Health Nutr..

[35] (2014). LARN Levels of Reference Intake of Nutrients and Energy for the Italian Population IV Revision-CRANUT. https://eng.sinu.it.

[36] Healthy Diet. https://www.who.int/news-room/fact-sheets/detail/healthy-diet.

[37] Catalani F., Gibertoni D., Lorusso G., Rangone M., Dallolio L., Todelli S., Lorenzini A., Tiso D., Marini S., Leoni E. Consumption and adequacy of breakfast over a week in a sample of primary school children. Proceedings of the 51 Congresso Nazionale Societa Italiana di Igiene Abstract Book.

[38] Lauria L., Spinelli A., Buoncristiano M., Nardone P. (2019). Decline of childhood overweight and obesity in Italy from 2008 to 2016: Results from 5 rounds of the population-based surveillance system. BMC Public Health.

[39] Department for Veterinary Public Health (2010). Nutrition and Food Safety Directorate-General for Food Safety and Nutrition National: Guidelines for School Restaurants. Italy. https://www.salute.gov.it/imgs/C_17_pubblicazioni_1248_allegato.pdf.

[40] R: The R Project for Statistical Computing. https://www.r-project.org/.

[41] Kawalec A., Pawlas K. (2021). Breakfast Frequency and Composition in a Group of Polish Children Aged 7–10 Years. Nutrients.

[42] Lauria L., Spinelli A., Cairella G., Censi L., Nardone P., Buoncristiano M. (2015). Dietary habits among children aged 8–9 years in Italy. Ann. Dell’istituto Super. Sanità.

[43] Whiting S., Buoncristiano M., Gelius P., Abu-Omar K., Pattison M., Hyska J., Duleva V., Milanović S.M., Zamrazilová H., Hejgaard T. (2021). Physical Activity, Screen Time, and Sleep Duration of Children Aged 6–9 Years in 25 Countries: An Analysis within the WHO European Childhood Obesity Surveillance Initiative (COSI) 2015–2017. Obes. Facts.

[44] Roccaldo R., Censi L., D’Addezio L., Toti E., Martone D., D’Addesa D., Cernigliaro A., Censi L., D’Addesa D., ZOOM8 Study Group (2014). Adherence to the Mediterranean diet in Italian school children (The ZOOM8 Study). Int. J. Food Sci. Nutr..

[45] Monsivais P., Aggarwal A., Drewnowski A. (2014). Time Spent on Home Food Preparation and Indicators of Healthy Eating. Am. J. Prev. Med..

[46] Troiano G., Severgnini M., Cirrincione M.L., Frittoli E., Firmi A.M., Clasadonte V. (2020). Improving quality in school canteens: Un fiore in mensa (A flower in canteen) project from ATS Val Padana (Italy). J. Prev. Med. Hyg..

[47] Ministero della Salute Linee di Indirizzo Nazionale Per la Ristorazione Collettiva. https://www.salute.gov.it/portale/nutrizione/dettaglioContenutiNutrizione.jsp?lingua=italiano&id=1648&area=nutrizione&menu=ristorazione.

[48] Cotwright C.J., Alvis C., Jimenez F.D.J., Farmer P., Okoli C., Delane J., Cox G.O. (2020). Improving Willingness to Try Fruits and Vegetables Among Low-Income Children Through Use of Characters. Health Equity.

[49] Zhang P., Lee J.E., Stodden D., Gao Z. (2019). Longitudinal Trajectories of Children’s Physical Activity and Sedentary Behaviors on Weekdays and Weekends. J. Phys. Act. Health.

[50] Sigmundová D., Sigmund E. (2021). Weekday-Weekend Sedentary Behavior and Recreational Screen Time Patterns in Families with Preschoolers, Schoolchildren, and Adolescents: Cross-Sectional Three Cohort Study. Int. J. Environ. Res. Public Health.

[51] Evenhuis I., Jacobs S., Vyth E., Veldhuis L., De Boer M., Seidell J., Renders C. (2020). The Effect of Supportive Implementation of Healthier Canteen Guidelines on Changes in Dutch School Canteens and Student Purchase Behaviour. Nutrients.

[52] Grimm K.A., Kim S.A., Yaroch A.L., Scanlon K.S. (2014). Fruit and vegetable intake during infancy and early childhood. Pediatrics.

[53] Caton S., Blundell-Birtill P., Ahern S., Nekitsing C., Olsen A., Møller P., Hausner H., Remy E., Nicklaus S., Chabanet C. (2014). Learning to eat vegetables in early life: The role of timing, age and individual eating traits. PLoS ONE.

[54] Wang Y., Cai L., Wu Y., Wilson R.F., Weston C., Fawole O., Bleich S.N., Cheskin L.J., Showell N.N., Lau B.D. (2015). What childhood obesity prevention programmes work? A systematic review and meta-analysis. Obes. Rev..

[55] An R. (2016). Weekend-weekday differences in diet among U.S. adults, 2003–2012. Ann. Epidemiol..

[56] Burchartz A., Oriwol D., Kolb S., Schmidt S.C., Wunsch K., Manz K., Niessner C., Woll A. (2021). Comparison of self-reported & device-based, measured physical activity among children in Germany. BMC Public Health.

