# Supplementary material for: Weekday and Weekend Differences in Eating Habits, Physical Activity and Screen Time Behavior among a Sample of Primary School Children: The “Seven Days for My Health” Project
Source: Int J Environ Res Public Health. 2022 Apr 1;19(7):4215. doi: 10.3390/ijerph19074215 (PMC8998659; doi:10.3390/ijerph19074215)
Supplement: Supplementary file 1 [file ijerph-19-04215-s001.zip › ijerph-1634748-supplementary.pdf]

Table S1.

	**Inadequate Snack**	**Adequate Snack**	***p*-Value**
**Less than 3 school-provided snack per week**	**37 (28%)**	**20 (6.7%)**	**<0.001**
**3 or more school-provided snack per week**	**93 (72%)**	**278 (93%)**	

Table S2.

**Characteristic**	**Inadequate Lunch**	**Adequate Lunch**	***p*-Value**
0	75 (38%)	16 (7.0%)	<0.001
1	124 (62%)	213 (93%)	
